# Acylated Inulin as a Potential Shale Hydration Inhibitor in Water Based Drilling Fluids for Wellbore Stabilization

**DOI:** 10.3390/molecules29071456

**Published:** 2024-03-24

**Authors:** Kaihe Lv, Haokun Shen, Jinsheng Sun, Xianbin Huang, Hongyan Du

**Affiliations:** Department of Petroleum Engineering, China University of Petroleum (East China), Qingdao 266580, China

**Keywords:** inulin, acylation, shale inhibitor, water-based drilling fluid, wellbore stability

## Abstract

Shale hydration dispersion and swelling are primary causes of wellbore instability in oil and gas reservoir exploration. In this study, inulin, a fructo-oligosaccharide extracted from Jerusalem artichoke roots, was modified by acylation with three acyl chlorides, and the products (C10-, C12-, and C14-inulin) were investigated for their use as novel shale hydration inhibitors. The inhibition properties were evaluated through the shale cuttings hot-rolling dispersion test, the sodium-based bentonite hydration test, and capillary suction. The three acylated inulins exhibited superb hydration-inhibiting performance at low concentrations, compared to the commonly used inhibitors of KCl and poly (ester amine). An inhibition mechanism was proposed based on surface tension measurements, contact angle measurements, Fourier-transform infrared analysis, and scanning electron microscopy. The acylated inulin reduced the water surface tension significantly, thus, retarding the invasion of water into the shale formation. Then, the acylated inulin was adsorbed onto the shale surface by hydrogen bonding to form a compact, sealed, hydrophobic membrane. Furthermore, the acylated inulins are non-toxic and biodegradable, which meet the increasingly stringent environmental regulations in this field. This method might provide a new avenue for developing high-performance and ecofriendly shale hydration inhibitors.

## 1. Introduction

Approximately 75% of drilled formations belong to shale formations, and over 90% of wellbore instability incidents occur during the process of drilling these shale formations [1,2,3]. Shale formations disperse and swell after contacting the water present in drilling fluids because shale is inherently water-sensitive [4]. This hydration-related dispersing and swelling that occurs during drilling operations can cause several problems, such as stuck pipes and tight holes [5,6,7,8], which can ultimately result in wellbore instability [2,9,10]. The most recent data shows that the economic loss caused by wellbore instabilities worldwide is $1 billion per year [6], making it one of the greatest challenges faced in oil and gas drilling operations.

Inhibiting shale hydration is an effective method to avoid this wellbore instability [11]. Because of their excellent hydration-inhibition, lubrication, and temperature-resistance properties, oil-based drilling fluids (OBDFs) have typically been utilized in drilling engineering [12]. However, OBDFs cannot meet the increasingly stringent environmental regulations, as they are non-biodegradable and harmful to the environment [13]. Compared with OBDFs, water-based drilling fluids (WBDFs) are relatively more cost-effective and environmentally friendly. However, the hydration-related dispersing and swelling of shale is exacerbated by the high water content in WBDFs [14]. Thus, high-performance shale hydration inhibitors (SHIs) have long been studied with the objective of improving the inhibitive performance of WBDFs. The most common SHIs are classified into three main types: potassium salts [15,16], ionic liquids [17,18], and amine polymers [19,20,21,22]. Huang et al. [23] prepared a hydrophobic cationic polyamine shale inhibitor (HCPA) by introducing hydrophobic groups into the molecular structure of amine shale inhibitors with branched chains. The results indicate that HCPA can significantly increase the water contact angle between rocks and mud cakes and reduce the water absorption capacity of clay and shale. The inhibitory performance is superior to commonly used shale inhibitors. Lv et al. [24] synthesized a hydrophobic cationic shale inhibitor ADD. The results indicate that ADD has good performance in inhibiting shale expansion and dispersion. This is because the extremity of ADD molecules can adsorb onto the surface of clay through electrostatic attraction and hydrogen bonding, and then, the hydrophobic non-polar end is exposed, forming a tight adsorption layer with high roughness and low surface free energy; Finally, the combination of electroneutralization and changes in wettability can alleviate shale hydration.

Although these SHIs exhibit satisfactory inhibition capabilities, they cannot satisfy all the engineering requirements in the drilling of complex formations. Moreover, the disadvantages of the above inhibitors, such as high cost, high toxicity, and poor temperature tolerance, limit their broader practical application [25]. Therefore, significant research on SHIs that are both high-performance and environmentally friendly is still necessary.

Certain natural materials and their derivatives exhibit great potential for inhibiting shale hydration. Several studies have revealed that natural surfactants can potentially serve as SHIs [26]. Moslemizadeh et al. [27] found that glycyrrhizin, extracted from the glycyrrhiza glabra root, has good inhibitory properties. Shadizadeh et al. [28] introduced the zizyphus spina-christi extract as the first plant-based SHI. Inspired by their work, we investigated the potential application of sugar-based surfactants (SBSs) as SHIs. There is strong interest in developing SBSs derived from renewable resources for potential application in industrial formulations [29]. Until now, the research has tended to focus on the derivatives of inulin, which is an abundant reserve polysaccharide found in chicory, onion, and garlic, with over 350,000 t produced annually [30]. Inulin consists of linear chains of β-2, 1-linked d-fructofuranose molecules terminated by a glucose residue through a sucrose-type linkage at the reducing end. It is modified by esterification, etherification, and carbamoylation in organic solvents using fatty acid methyl esters, alkyl epoxides, and alkyl isocyanates, respectively, to prepare surfactants [31,32,33] that have been applied as encapsulating and emulsification agents in surface coatings and pharmaceuticals. However, there are scarcely any reports about the use of inulin and its derivatives as SHIs in WBDFs.

In this study, we modified inulin using acyl chlorides to prepare three distinct non-ionic acylated derivatives with varying alkyl chain lengths (C10–C14) and evaluated their ability to inhibit shale hydration using immersion, shale cuttings hot-rolling dispersion, sodium-based bentonite (Na-bent) hydration, linear swelling, and capillary suction time tests. The inhibition mechanism was further investigated via particle size distribution measurement, X-ray diffraction (XRD), surface tension measurement, contact angle measurement, Fourier-transform infrared (FT-IR), and scanning electron microscopy (SEM).

## 2. Results and Discussion

### 2.1. Characterization

**Figure 1:**
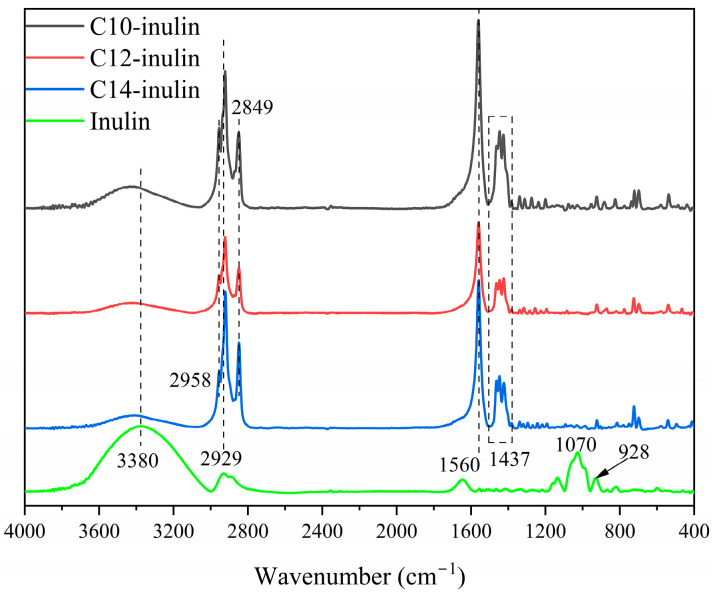
FT-IR spectra of inulin and acylated inulins.

Figure 1 shows the FT-IR spectra of untreated inulin and the acylated inulin samples used in this work: C10-inulin, C12-inulin, and C14-inulin. Inulin has a broad peak centering at 3380 cm^−1^ from the O–H stretching of associated glucose and fructose units in the polysaccharide backbone. The band around 2929 cm^−1^ corresponds to C–H stretch, and the peak at 1560 cm^−1^ can be assigned to the hydroxyl bending mode. The bands at 1070 cm^−1^ and 928 cm^−1^ correspond to C–O–C stretching, respectively [34] The spectra of acylated inulins exhibit some emerging peaks at 1560 cm^−1^ and 1437 cm^−1^, which are attributed to the asymmetric C=O stretching and ester carbonyl stretching, respectively, present within the carbonyl ester group [35]. The absorption peaks at 2958 cm^−1^ and 2849 cm^−1^ are attributed to asymmetric CH_3_ stretching and symmetric CH_2_ stretching, respectively [36]. Altogether, the results of FT-IR indicate that the inulin has been modified successfully.

**Figure 2:**
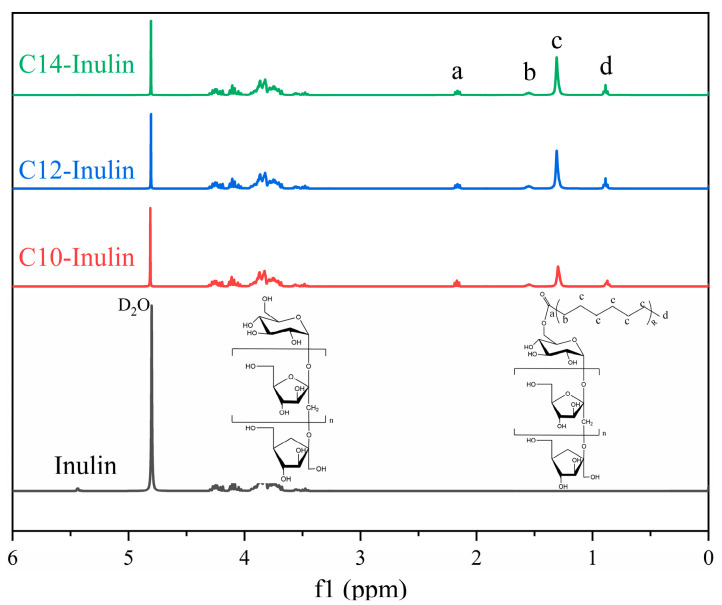
^1^H NMR spectra of inulin (a), C10-inulin (b), C12-inulin, (c) and C14-inulin (d).

Inulin and acylated inulins were also characterized by ^1^H NMR, and these spectra are shown in Figure 2. The prominent peak at 4.80 ppm is from the solvent (D_2_O) [37]. It is well-known that the peaks at 3.0–4.5 ppm can be assigned to the absorption peaks of protons in the fructose and glucose in the skeleton of inulin [38,39,40]. Additionally, the peaks at 5.4 ppm are assigned to the α-anomeric forms of free glucose [40]. The ^1^H NMR signals from 0.8–0.9 ppm, appearing as a triplet, signify the three protons of the terminal methyl group of the acyl chain. Peaks a at 2.10–2.12 ppm, peaks b at 1.50–1.60 ppm, and peaks c at 1.30 ppm are all related to the methylene group. The peaks a are close to those expected for the ester bond, indicating successful esterification. After comparing the ^1^H NMR spectra for acylated inulin with that of original, untreated inulin in the same solvent D_2_O, it is evident that acylation has occurred. Thus, the ^1^H NMR spectra further confirm the successful synthesis of acylated inulins.

### 2.2. Inhibition Evaluation

#### 2.2.1. Shale Cuttings Hot-Rolling Dispersion Test

**Figure 3:**
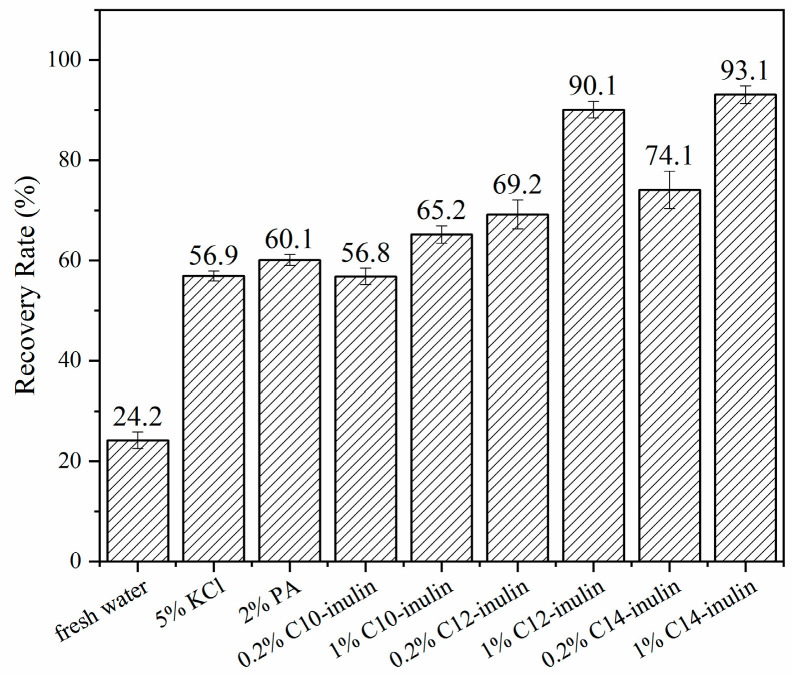
Shale cuttings hot-rolling recovery rate in water and different inhibitor solutions.

Shale cuttings are clay-rich detritus that are generated during the drilling process, and the hot-rolling dispersion test simulates the process that shale cuttings undergo throughout the drilling process [25]. Figure 3 shows the recovery rate of shale cuttings in both water and various inhibitor solutions that were aged at 120 °C. The lowest recovery rate of 24.2%, indicating that the shale cuttings dispersed easily, was observed in water. Comparatively, the recovery rates of shale cuttings in 5% KCl and 2% PA were much higher, and the low-concentration (0.2%) acylated inulin solutions showed similar inhibition capacities to these solutions, with values between 56.8% and 74.1%. With an increased concentration of 1%, much higher recovery rates of 90.1% and 93.1% for C12-inulin and C14-inulin, respectively, were obtained, demonstrating that the inhibition performance of acylated inulin is positively correlated with its concentration. Furthermore, the three acylated inulin samples exhibited different inhibition capacities. The recovery rates of 1% acylated inulin samples improved from 65.2% to 93.1% when the chain length was increased from C10 to C14, indicated that the inhibition performance of acylated inulin is related to the length of the alkyl chain.

#### 2.2.2. Na-Bent Hydration Test

**Figure 4:**
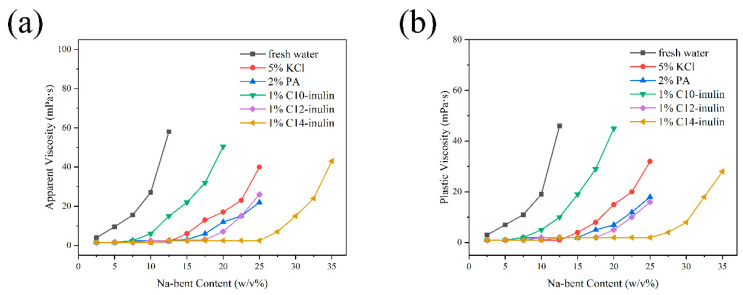
Variations of AV (**a**) and PV (**b**) as a function of Na-bent content in different inhibitor solutions.

Investigating the influence of Na-bent on the rheological properties of WBDFs with different inhibitors is considered an effective method for evaluating the inhibitive capacity of SHIs. Clay minerals dispersed into plate-like particles in either water or weak-inhibition systems and combine together to form “house of card” structures that significantly increase the WBDF viscosity [41]. As shown in Figure 4, the AV (Figure 4a) and PV (Figure 4b) increased gradually with the addition of Na-bent for all tested solutions. High viscosity values at low concentrations of Na-bent loading were observed in water, whereas the inhibitors clearly delayed the viscosity increase. This indicates that the degree of hydration dispersion is reduced. Specifically, a 1% C14-inulin solution produced the lowest values of AV (18 mPa∙s) and PV (7.5 mPa∙s) at a 30 *w*/*v*% of Na-bent loading, indicating that the C14-inulin could inhibit the hydration dispersion of Na-bent more effectively than the concentrated solutions of 5% KCl and 2% PA.

#### 2.2.3. Capillary Suction Time Test

**Figure 5:**
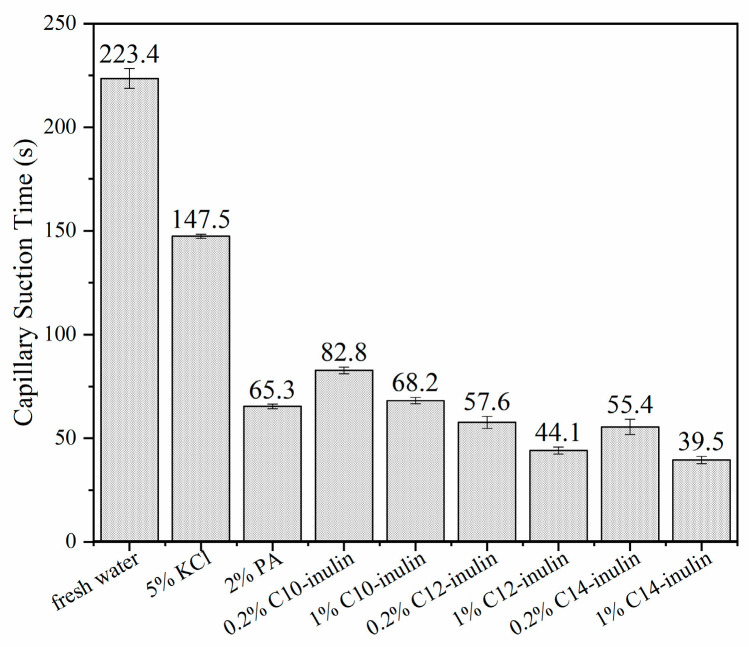
Capillary suction time of Na-bent dispersions in water and varied SHI solutions.

The CST tests were conducted to evaluate the capacity of SHIs to inhibit the hydration dispersion of Na-bent. Na-bent would hydrate and disperse into fine particles in water, which would consume free water by absorption. When the dispersion contacts the filter paper, the free water can permeate along the filter paper, while the fine particles would stack tightly on the paper, resulting in a low-permeability filter cake that prevents the further permeation of water. A low free water content and filter cake permeability correspond to high CST values. Figure 5 shows the CST values of Na-bent dispersions in fresh water and a variety of inhibitor solutions. The CST of Na-bent in fresh water is 223.4 s, which is much higher than that of any of the other inhibitor solutions, indicating that Na-bent is easily hydrated and dispersed in water. Compared with water, the CSTs of Na-bent in 5% KCl (147.5 s) and 2% PA (65.3 s) are much shorter, and the low-concentration acylated inulin solutions show similar inhibitive capacities, with CST values between 55.4 s and 82.8 s. With an increased concentration of 1% acylated inulin, even shorter CST values of 44.1 s and 39.5 s are obtained by the C12-inulin and C14-inulin solutions, respectively, demonstrating that the inhibition property of acylated inulin is positively correlated with the concentration of acylated inulin. In addition, the three acylated inulins exhibited varying inhibitive capacity, with the CST of 1% acylated inulin decreasing from 68.2 s to 39.5 s when the alkyl chain length increased from C10 to C14, which verifies that the inhibition capacity of acylated inulin is related directly to the alkyl chain length.

#### 2.2.4. Particle Size Distribution

**Figure 6:**
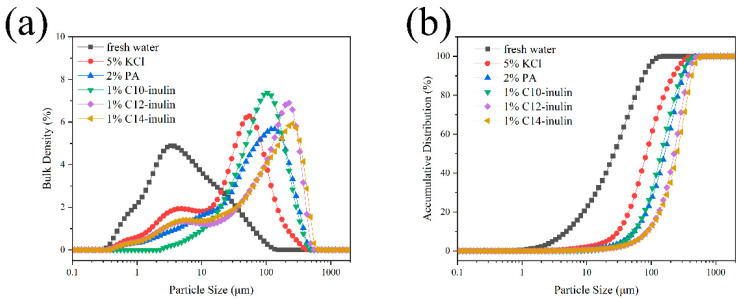
Particle size distribution of Na-bent dispersion with different inhibitors: (**a**) Particle size differential distribution curve, (**b**) Particle size cumulative distribution curve.

After the Na-bent hydration test, the particle size of each sample was measured to confirm that the hydration dispersion of Na-bent was inhibited by adding SHIs. The differential and accumulative size distribution of Na-bent dispersion are shown in Figure 6. The size of Na-bent in water is much smaller than in the inhibitor solutions, indicating that the hydration dispersion of Na-bent was inhibited effectively by the SHIs. The median size (D_50_) of Na-bent particles in water, 5% KCl, 2% PA, 1% C10-inulin, 1% C12-inulin, and 1% C14-inulin was 26 μm, 84 μm, 145 μm, 162 μm, 224 μm, and 262 μm, respectively, with the increasingly large particle size reflecting the increasing inhibition performance of the SHIs. The trends observed here coincide with the results of the Na-bent hydration test (Figure 4) and CST test (Figure 5).

#### 2.2.5. Immersion Test

**Figure 7:**
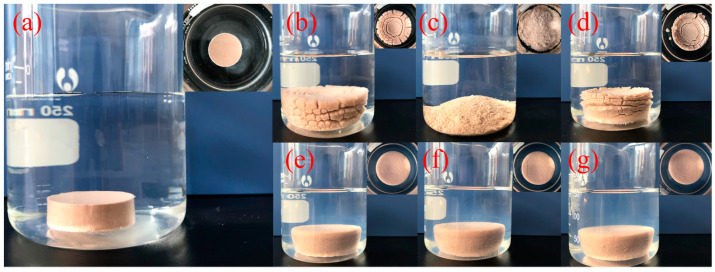
Na-bent columns immediately after immersion began (**a**) and after 12 h immersion in water (**b**), 5% KCl solution (**c**), 2% PA solution (**d**), 1% C10-inulin solution (**e**), 1% C12-inulin solution (**f**), 1% C14-inulin solution (**g**). The inset in each panel provides a top view of the column.

The immersion test results are shown in Figure 7. Figure 7a presents the original state of the Na-bent column in water. Images of the Na-bent columns after immersion in different inhibitor solutions for 12 h are shown in Figure 7b–g. Notably, after being immersed in water for 12 h, the surface of the Na-bent column softened and cracked (Figure 7b), and the column swelled in size. When immersed in 5% KCl, the Na-bent column could not maintain structural integrity and collapsed entirely within 30 min, but the Na-bent column still showed little sign of hydration (Figure 7c). For PA (Figure 7d), both swelling and dispersion were less severe compared with water immersion. Additionally, all three acylated inulin immersions exhibited excellent inhibition properties. After being immersed for 12 h, the Na-bent columns largely maintained their original form (Figure 7e–g), and the column size increased only slightly.

#### 2.2.6. Linear Swelling Test

**Figure 8:**
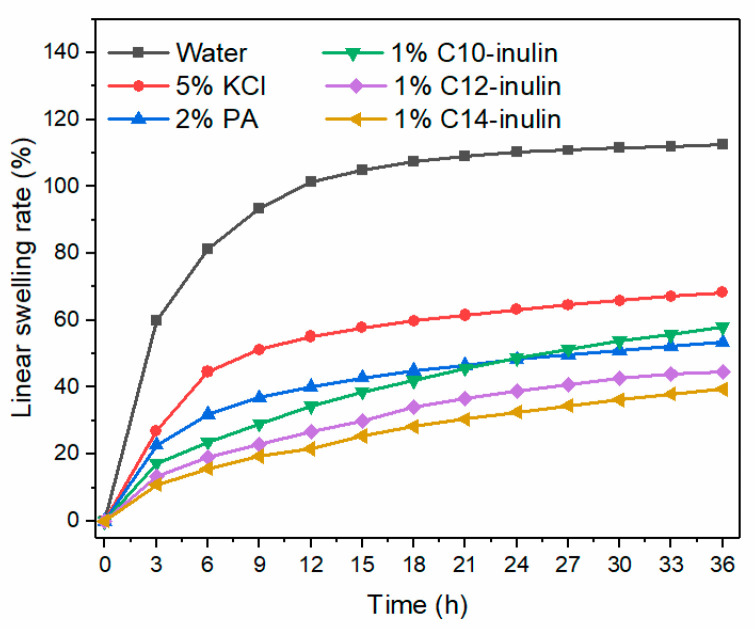
Linear swelling rates of Na-bent volumes in water and different inhibitor solutions.

To quantify the swelling, linear swelling tests were conducted to further assess the ability of inhibitors to prevent Na-bent columns from swelling. These results are shown in Figure 8. The swelling curves of all samples displayed a similar tendency to increase sharply in the first 6 h and then follow a relatively moderated increase for the remainder of the experiment. The Na-bent columns swelled drastically in water, with a 102% linear swelling rate after 36 h of immersion. Compared with water, the SHIs significantly reduced the column swelling. In particular, the linear swelling rate of the Na-bent column in the C14-inulin solution was the lowest at 37.5%, confirming that 1% C14-inulin was most effective in inhibiting the hydration swelling of the Na-bent column. Notably, the linear swelling rate of the Na-bent column in a 1% C10-inulin solution was higher than that in a 2% PA solution after the 36 h immersion, which implies that the long-term inhibition ability of the C10-inulin, with respect to the Na-bent dispersion, is inferior to that of PA at the concentrations evaluated.

#### 2.2.7. XRD Analysis

**Figure 9:**
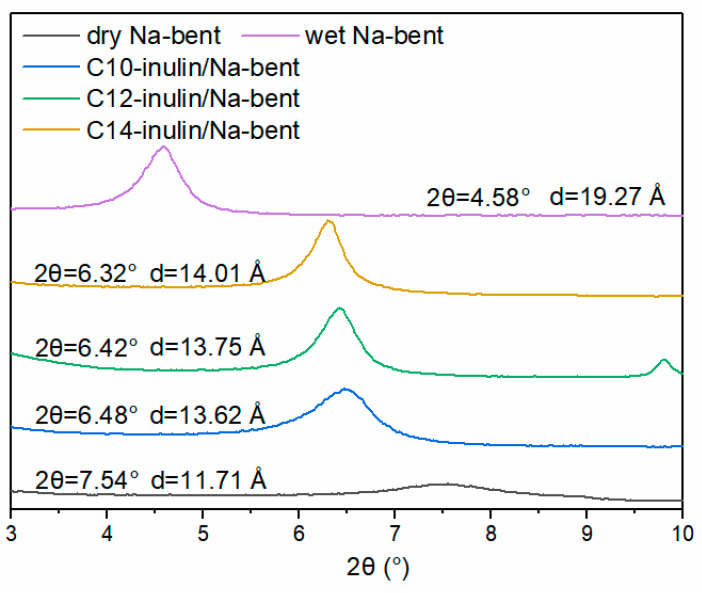
XRD patterns of dry Na-bent, wet Na-bent, and modified Na-bent, with different inhibitors.

To further understand the swelling behavior of Na-bent in different inhibitor solutions on a microscopic level, XRD patterns and interlayer spacing (d_001_) of original, dry Na-bent, and Na-bent in either water or inhibitor solutions are shown in Figure 9. For the original dry Na-bent, the interlayer spacing was 11.71 Å. In the case of Na-bent in water, a high d_001_ value of 19.27 Å was obtained, reflecting the high degree of hydration swelling that was previously observed. Contrarily, the interlayer spacing of Na-bent in the acylated inulin solutions is much smaller, which demonstrates the strong anti-swelling capacity that these SHIs impart to the clay mineral. It is worth noting that the interlayer spacing of Na-bent in the 1% acylated inulin solutions increased with increasing alkyl chain length, implying that the acylated inulin intercalated into the interlayer of Na-bent because the long alkyl chain is incompatible with the water surroundings and forms some repulsion, which is more impactful for samples with longer alkyl chains.

### 2.3. Inhibitive Mechanism Investigation

#### 2.3.1. Static Surface Tension Measurement

**Figure 10:**
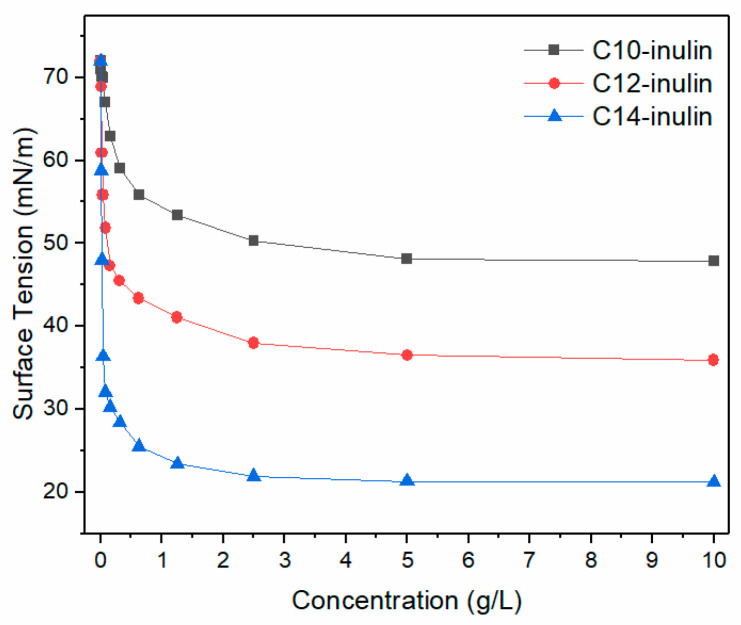
Variation in surface tension with increasing concentration of the three acylated inulins.

Shale is a hydrophilic rock that contains a large number of micro-pores. Therefore, capillary phenomena occur easily upon contact with water, which accelerates shale hydration. According to the Laplace formula (∆p=2σ/R), where R represents radius of micro-pores, the capillary force (∆p) is directly proportional to the surface tension (σ). The surface tension of the three acylated inulin solutions with different concentrations was measured (Figure 10), and it was found that the surface tension declined with increasing inulin concentration. When the concentration reached 10 g/L, the maximum concentration evaluated, the surface tensions of C10-inulin, C12-inulin, and C14-inulin decreased from their initial values, which exceeded 70 mN/m, to 50.1 mN/m, 38.3 mN/m, and 20.9 mN/m, respectively. The low surface tension, especially of the C14-inulin solution, made water invasion more difficult, resulting in the remarkable anti-dispersion and anti-swelling properties that the shale cuttings displayed in its solution, as depicted in Figure 3 and Figure 8, respectively.

#### 2.3.2. Contact Angle Measurement

**Figure 11:**
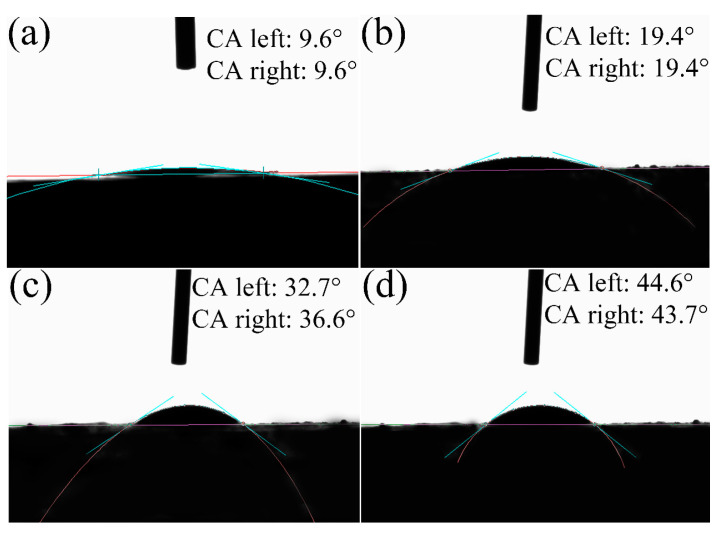
Contact angel between deionized water and original shale (**a**), C10-inulin-modified shale (**b**), C12-inulin-modified shale (**c**), and C14-inulin-modified shale (**d**).

Altering the wettability of shale from hydrophilic to hydrophobic is a desirable method for improving its stability because wellbore instability is directly related to the physicochemical interaction between wellbores and drilling fluid [17]. The contact angle (CA) is commonly used to characterize material wettability, and these data were obtained in this study for both the original shale slices and shale slices modified with the three acylated inulins, as shown in Figure 11. Compared with the 9.6° CA of the original shale, the CAs of modified shale slices increased, indicating that the shale slices are more hydrophobic. In addition, the CAs of the modified shale increased with the increasing alkyl chain length of the acylated inulins, implying that stronger hydrophobicity can be attributed to longer alkyl chains. During the process of water invasion, the acylated inulins with long alkyl chains were adsorbed onto the shale surface and inserted into the clay interlayers, which enhanced the hydrophobicity of shale and subsequently hindered water ingress, thus successfully inhibiting the shale hydration.

#### 2.3.3. FTIR Analysis

**Figure 12:**
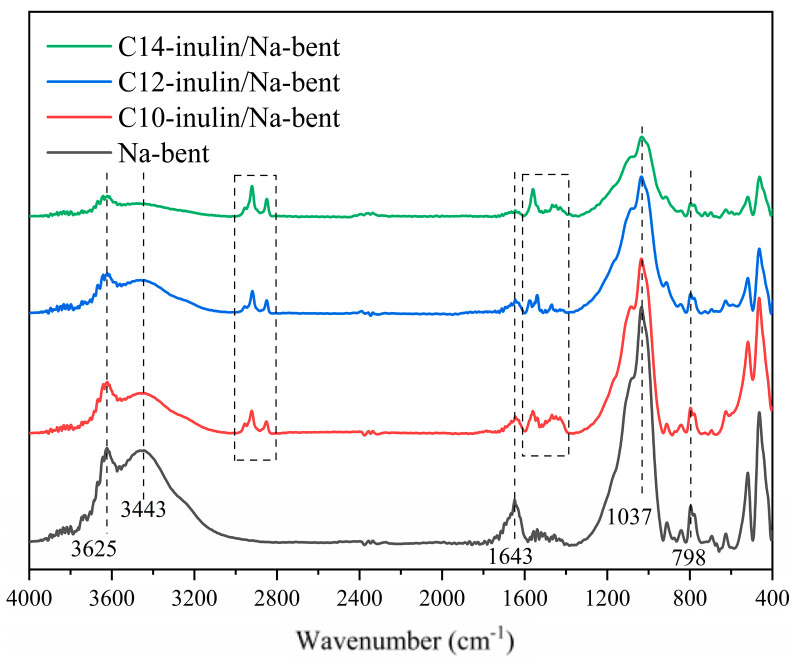
FT-IR spectra of Na-bent and acylated inulin/Na-bent hybrids.

To gain insight into the adsorption mechanism of acylated inulins on Na-bent, the FT-IR spectra of the original Na-bent and Na-bent hybrids modified with the various acylated inulins are shown in Figure 12.

The absorption peaks of the original Na-bent at 3625 cm^−1^, 1037 cm^−1^, 916 cm^−1^, and 798 cm^−1^ are attributed to O–H stretching vibration, asymmetric Si–O stretching vibration, Al-OH stretching vibration, and symmetric Si–O stretching vibration, respectively. Further, the absorption peaks at 3443 cm^−1^ and 1643 cm^−1^ are attributed to physisorbed water stretching vibration and the water deformation band, respectively [42].

In the Na-bent/acylated inulins’ hybrid spectra, some newly emerged peaks, which can be assigned to acylated inulin, were observed. The new stretching bonds of alkane confirm the incorporation of acylated inulin into the Na-bent. Compared with original Na-bent, the peak of the bending bond of Si–O (1037 cm^−1^) is red-shifted and broadened, which indicates the hydrogen bonds formed between Na-bent and acylated inulins [42].

#### 2.3.4. Morphology

**Figure 13:**
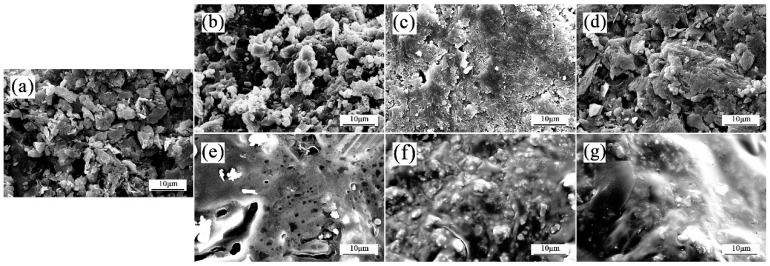
SEM images of an original dried shale sample (**a**) and shale samples in water (**b**) and different inhibitor solutions: 5% KCl (**c**); 2% PA (**d**); 1% C10-inulin (**e**); 1% C12-inulin (**f**); and 1% C14-inulin (**g**).

The surface morphology of shale cuttings, both before and after hot rolling at 120 °C in water and other inhibitor solutions, was analyzed using SEM. The original surface of the dried shale cuttings was compact and smooth (Figure 13a), whereas after hot rolling in water (Figure 13b), the distributed clay particles and large amount of pores on the shale surface implied that the shale had been significantly hydrated. Similarly, though less significant, phenomena clearly occurred in the 5% KCl solution (Figure 13c) and 2% PA solution (Figure 13d). These visible pores facilitate water invasion, resulting in more severe shale dispersion and ultimately wellbore instability. Notably, a smooth membrane has formed on the shale surface in all the acylated inulin solutions. In accordance with the hot-rolling dispersion test results (Figure 3), more pores are visible in the surface of the shale cuttings after hot rolling in C10-inulin (Figure 13e) compared to C12 or C14. Contrarily, the smooth membrane is most obvious for the C12- and C14-inulin samples (Figure 13f,g). The SEM images directly demonstrate the remarkable capacity of the three acylated inulins to inhibit shale hydration.

#### 2.3.5. Probable Inhibition Mechanism

**Figure 14:**
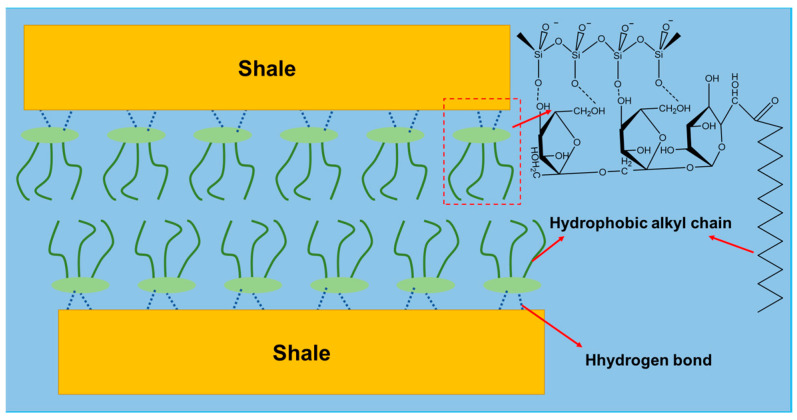
Schematic of inhibitory mechanism of hydrophobically modified inulin.

The experimental and mechanistic analysis of the inhibition properties of acylated inulin has been thoroughly investigated and presented thus far. According to these results and evaluations, the probable inhibition mechanism has been revealed as follows. Acylated inulin belongs to a group of sugar-based surfactants that can significantly reduce the surface tension of water, leading to low capillary forces and subsequently slowing the invasion of water into shale formations. The molecular structure of acylated inulin contains both hydrophilic and hydrophobic parts. The hydrophilic hydroxyl end of the inulin combines with the Si–O bond on the shale surface via hydrogen bonding, so the acylated inulin can adsorb onto the shale surface and form a compact sealed membrane. This hydrophobic membrane increases the hydrophobicity of the shale surface, thus preventing water from contacting the shale formation and ultimately reducing the hydration potential of the shale. The schematic of this inhibitory mechanism performed by hydrophobically modified inulin is illustrated in Figure 14.

#### 2.3.6. Environmental Aspects

**Figure 15:**
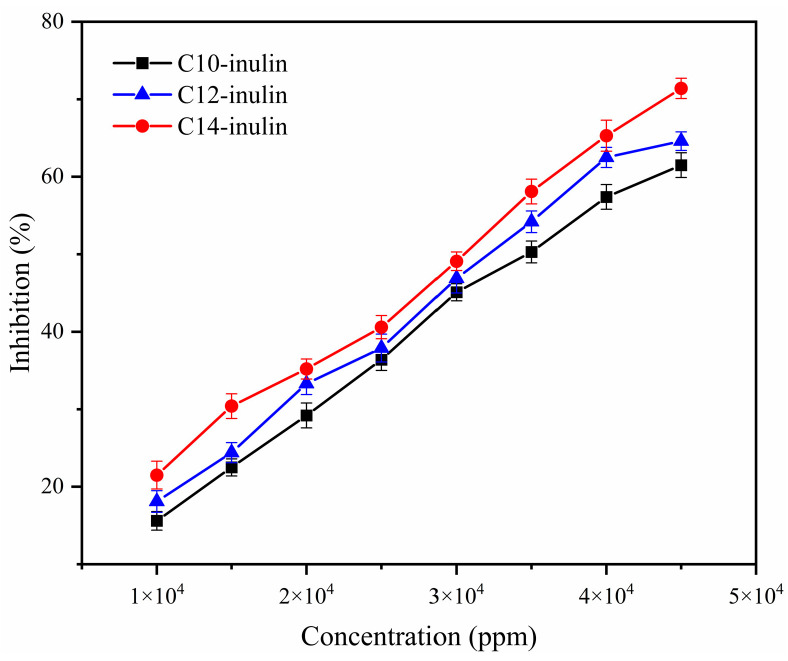
Variation of inhibition with increasing concentration of the three acylated inulins.

Figure 15 shows the ecotoxicological results of *Vibrio fischeri* bioassays conducted with the acylated inulin solutions at varied concentrations. For clarity, the results were analyzed mathematically, as summarized in Table 1. For all three acylated inulin solutions, the inhibition effect increased with increasing inulin concentration, which implies that inulins at higher concentrations will exhibit higher toxicities. Moreover, the toxicity of the three acylated inulins is related to the alkyl chain length, as the C14-inulin clearly shows higher toxicity than the C10-inulin. However, the EC_50_ values of acylated inulins are still higher than standard values (>30,000 ppm, a concentration is considered non-toxic), signifying that they are non-toxic [26]. Furthermore, considering that common usage concentrations are between 10,000–30,000 ppm, all three of the acylate inulin SHIs can be regarded as non-toxic.

To evaluate the biodegradability of the three acylated inulins, the COD and BOD5 of a 1% solution of C10-inulin, C12-inulin, and C14-inulin were measured. As shown in Table 2, the biological oxygen demand and chemical oxygen demand of the three acylated inulin meet the pollutant emission standards set by the petrochemical industry (GB-31571-2015). The ratio of the three types of acylated inulin is greater than 1, indicating that all three types of acylated inulin are biodegradable.

## 3. Materials and Methods

### 3.1. Materials

Inulin (degree of polymerization < 9) was purchased from Sinopharm Chemical Reagent Co., Ltd., Shanghai, China. The inulin was dried at 70 °C for 24 h before use. Decanoyl chloride (C10; 98%), lauroyl chloride (C12; 98%), and myristoyl chloride (C14; 98%) were purchased from Sinopharm Chemical Reagent Co., Ltd., China. Potassium chloride (KCl; 99.5%) and poly (ester amine) (PA; 99%) were purchased from Aladdin Industrial Co., Ltd., Shanghai, China. All chemicals were used without further purification.

Na-bent was obtained from J&K Scientific Ltd., Beijing, China. The shale samples were obtained from the Sichuan Province in China. The mineral composition of the shale samples is shown in Table 3.

### 3.2. Acylated Inulin Preparation

**Figure 16:**
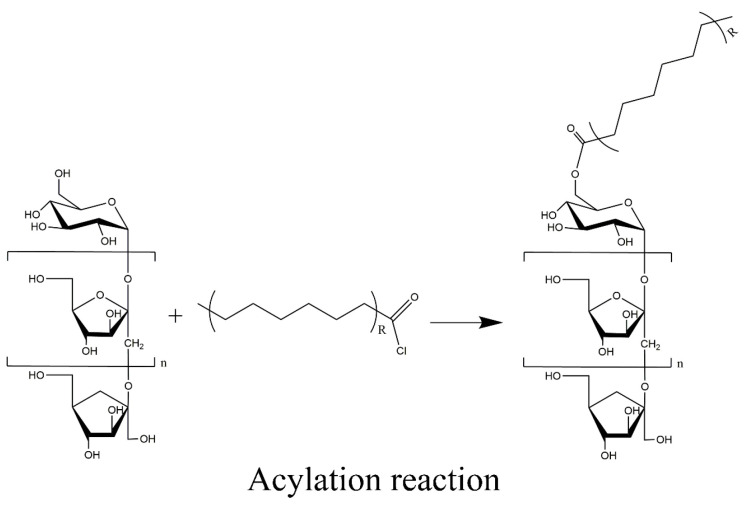
Fabrication of acylated inulin. (R: 8, 10, or 12).

A series of acylated inulin was synthesized according to the method proposed by Williams et al. [29]. Inulin (10 g, 61.70 mmol) was added to 50 mL of NaOH solution (20 *w*/*v*%), which was stirred at room temperature. The solution was then loaded into a reaction flask. Next, decanoyl chloride (1 equiv, 61.70 mmol, 12 mL), lauroyl chloride (1 equiv, 61.70 mmol, 13.77 mL), or myristoyl chloride (1 equiv, 61.70 mmol, 15.54 mL) was added to the reaction vessel using a double-channel microinjection pump (WZS-50F6, Hangzhou, China) with an injection speed of 0.2 mL/min. The molar ratio of acyl chloride and inulin was 1:1 for all samples. The reaction was allowed to continue until the modified products precipitated out of the solution. The precipitation was completed over 2 h. The solid products were recovered by freeze-drying and were washed using a Soxhlet extraction with cyclohexane for 12 h to completely remove any unreacted fatty acid acyl chlorides. The products were then dried in a vacuum dryer at 70 °C until the weight was constant. The final products were then coded according to the length of their alkyl chain: C10-inulin, C12-inulin, and C14-inulin. The schematic of the synthesis mechanism is shown in Figure 16.

### 3.3. Inhibition Property Evaluation

#### 3.3.1. Shale Cuttings Hot-Rolling Dispersion Test

To simulate the condition of shale cuttings in the drilling process, shale cuttings hot-rolling dispersion tests were conducted [43,44]. First, the shale cuttings samples were sieved through 6–10 mesh before being dried at 103 °C for 10 h in a drying oven. Next, shale samples (50 g each) were added to 350 mL of either fresh water or one of the inhibitor solutions in an aging cell, which were then rolled in a roller oven at 120 °C for 16 h. After the test was completed, the cells were cooled to room temperature. The remaining shale cuttings were collected and re-dried at 103 °C for 24 h in a drying oven. The re-dried shale cuttings were sieved through a 40 mesh sieve, and the recovered shale cuttings were weighed to obtain the mass $M_{i}$. The recovery rate, $R_{i}$, of shale cuttings after hot-rolling was then calculated using the following equation:

$$R_{i}\left(\%\right)=\frac{M_{i}}{50}\times 100\%$$

#### 3.3.2. Na-Bent Hydration Test

As shown in Table 3, clay minerals are the main components of the shale samples, and the illite/smectite mixed layer of clay mineral is prone to hydration. Therefore, the inhibition performance of the SHIs on the clay minerals specifically was evaluated by the Na-bent hydration test. Different amounts of Na-bent, ranging between 10 and 100 g, were added into 400 mL of either fresh water or inhibitor solutions, and the mixture was stirred for 30 min at 10,000 rpm. The mixture was then loaded into an aging cell and hot-rolled in a roller oven at 120 °C for 16 h. After the test was completed, the cells were cooled to room temperature. The viscosity of the dispersion at room temperature was tested using a six-speed rotational viscometer (ZNN-D6, Qingdao Haitongda, Qingdao, China). The reading value of the viscometer at rotation rates of 600 and 300 rpm ($\theta_{600}$, $\theta_{300}$) was recorded. Equations (1) and (2) were used to calculate the apparent viscosity (*AV*) and plastic viscosity (*PV*), respectively, according to the API standard [44].

(1) $$AV=\theta_{600}/2(\mathrm{mPa}\cdot i)$$

(2) $$PV=\theta_{600}-\theta_{300}(\mathrm{mPa}\cdot i)$$

After the Na-bent hydration test, a portion of the dispersion was retained and marked for particle size distribution measurements. The remaining dispersion was centrifuged at 8000 rpm for 20 min and washed several times with deionized water until the upper liquid became clear. Then, the precipitate was dried at 103 °C for 24 h and ground to powder for FT-IR and XRD analyses.

#### 3.3.3. Capillary Suction Time Test

**Figure 17:**
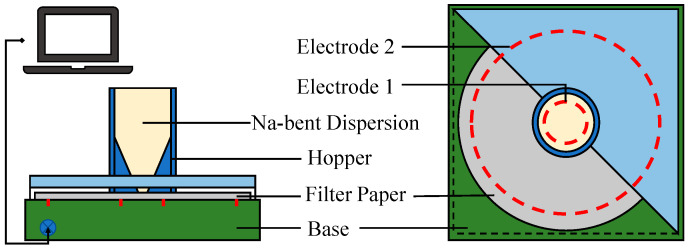
Schematic diagram of capillary suction time tester.

The capillary suction time (CST) test was conducted to simulate free water from WBDF penetrating into the shale formation under capillary suction pressure. CST testing is a method of measuring the time required for various testing fluids to infiltrate specialized filter paper within a certain distance when mixed with shale powder. This test is widely used to evaluate the inhibitory performance of the test fluid in terms of hydration. The CST value represents the hydration inhibition characteristics of the additive. The stronger the inhibitory performance of the test fluid, the weaker the dispersibility of shale, resulting in a shorter time required for the slurry to pass through the specified distance. The schematic diagram of the capillary suction time tester is shown in Figure 17. In this test, 50 g of Na-bent was added into 400 mL of fresh water and inhibitor solutions, and the mixture was stirred for 30 min at 10,000 rpm. Then, 3 mL of each dispersion was injected into a special hopper for the CST test. Beginning in the center of the filter paper, the free water in the dispersion would then spread along it. When the water reached electrode 1, the system began timing the run and ended the timing when the water touched electrode 2. The resulting time interval is the CST.

#### 3.3.4. Immersion Test

**Figure 18:**
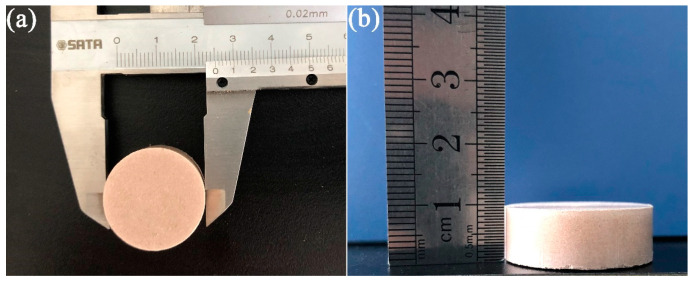
Top view (**a**) and side view (**b**) images of the Na-bent column.

The immersion test is a simple and intuitive method used to evaluate the inhibition performance of SHIs [33]. First, Na-bent columns were prepared by compressing 10.0 g of Na-bent into compact round cakes with a diameter and height of 2.5 and 1.0 cm, respectively (Figure 18). These cakes were compressed under 10 MPa pressure for 5 min. The Na-bent columns were then immersed in 200 mL of either water or inhibitor solutions, and the statuses of the immersed Na-bent columns were observed after 12 h.

#### 3.3.5. Linear Swelling Test

This method was designed to simulate the shale formation expansion caused by water invasion during the drilling process. Na-bent columns were prepared by the same method described in Section 3.3.4, and their initial heights ($H_{0}$) were measured. Then, the Na-bent columns were immersed in fresh water and inhibitor solutions in a linear swell meter. The height ($H_{1}$) at different times was recorded by the equipment automatically over the entire test time of 36 h. The linear swelling rate ($R_{l}$) was then calculated using the following equation:

$$R_{l}\left(\%\right)=\frac{H_{1}-H_{0}}{H_{0}}\times 100\%$$

#### 3.3.6. Ecotoxicological Test

*Vibrio fischeri* acute toxicity tests were performed using a Microtox^®^ analyzer (Model FX, Modern Water, London, UK), in accordance with both the manufacturer’s instructions and the ISO 11348-3: 2007 standard method. This test is based on the inhibition of the luminescence that is naturally emitted by the bacterium *Vibrio fischeri* after exposure to an SHI sample. The difference in light output, which is measured after a 30 min exposure, between the sample and blank (saline solution, 20 g/L NaCl), can be attributed to the effect that the SHI sample has on the organisms. This effect, reported as a % inhibition, is calculated by Microtox calculation software (SDIX 500).

The lyophilized freeze-dried *Vibrio fischeri* bacteria (strain NRRL B-11177) and reconstitution solution for the bacteria were purchased from Ecotox LDS s.r.l. (Milan, Italy). Before using the lyophilized bacteria, they were rehydrated with the reconstitution solution to produce a ready-to-use suspension. An osmotic adjustment solution (22% *w*/*v* NaCl, prepared in Milli-Q water) was used to bring the salinity of the samples to approximately 2% NaCl, which is optimal for *Vibrio fischeri*. After the preliminary tests, the concentration for 50% of maximal effect (EC_50_) values of the acylated inulin solutions were evaluated. Based on the data of inhibition rate and concentration, a set of regression equations and correlation coefficients for the experiment were fitted, with the concentration value corresponding to EC_50_ at an inhibition rate of 50%.

#### 3.3.7. Biodegradability Test

To evaluate SHI biodegradability, chemical oxygen demand (COD) was evaluated using the fast digestion spectrophotometric method (HJ/T 399-2007). The biochemical oxygen demand in five days (BOD_5_) was assessed via the microbial electrode method (HJ/T 86-2002). The biodegradability was evaluated by dividing the BOD_5_ by the COD.

### 3.4. Characterization Methods

#### 3.4.1. Fourier-Transform–Infrared (FT-IR) Spectroscopy

The FT-IR spectra of the acylated inulins were obtained using the KBr pellets method, where 0.9–2 mg of the sample was mixed in an agate mortar with 300 mg of KBr. Transparent pellets were obtained under vacuum (approximately 1 Torr) at 14 tons/cm^2^. The sample FT-IR spectra were recorded in the range of 4000–400 cm^−1^ using a FT-IR spectrometer (IRTracer-100, Shimadzu Corporation, Kyoto, Japan). The FT-IR spectra of the precipitate obtained from the Na-bent hydration test were recorded using the same parameters.

#### 3.4.2. ^1^H NMR Nuclear Magnetic Resonance (NMR)

The ^1^H NMR spectra of the acylated inulins were recorded using a Bruker Advance DPX-300 spectrometer (Bruker, Mannheim, Germany) with a 30° pulse at 25 °C. Samples (5 mg) were dissolved in 0.7 g of D_2_O and then loaded into NMR tubes for measurement.

#### 3.4.3. Scanning Electron Microscopy (SEM)

After the shale cuttings hot-rolling dispersion test, the shale samples were dried at 103 °C for 24 h in a vacuum-drying oven. The samples were then coated with gold using a sputter coater (SuPro Instruments ISC150, Shanghai, China), so that the surface morphology could be observed using SEM (Hitachi S4800, Hitachi City, Japan) at a 20 kV voltage.

#### 3.4.4. X-ray Diffraction (XRD)

After the Na-bent hydration test, the dried precipitate samples were analyzed by XRD. The XRD patterns were scanned with an X’Pert PRO MPD diffractometer over a range of 3° < 2*θ* < 10°, with a goniometric rate of 0.1 and an integration time of 0.4 s, and then, the backgrounds of the diffraction patterns were subtracted. The scan mode was continuous, using Cu Kα1 radiation (λ = 1.5406 Å). The voltage was 40 kV, and the tube current was 40 mA. The interlayer spacing of the Na-bent samples was calculated using Bragg’s equation [43].

#### 3.4.5. Particle Size Distribution

The particle size distribution of dispersion samples obtained after the Na-bent hydration test was determined using a Malvern Mastersizer 3000 particle size analyzer (Malvern, UK). The measurements were made at 25 °C, using disposable sample cells at a detection angle of 90°. The concentration of all samples was diluted to approximately 10.0 g/L with deionized water.

#### 3.4.6. Static Surface Tension Measurement

The static surface tension of the different acylated inulin solutions with various concentrations was measured with a dynamic contact angle meter and tensiometer (Data Physics, Filderstadt, Germany) by the Wilhelmy method. The solution was stirred for 1 min before the measurement of the surface tension, which was performed over a period of 180 s. The temperature was kept constant at 25 °C during all measurements. Before each measurement, the Pt-plate was cleaned and heated to red-hot in a gas flame. All measurements were repeated three times.

#### 3.4.7. Contact Angle Measurement

Contact angle is an important parameter to appraise surface wettability alterations, which can be a good strategy for evaluating shale stability. Air–water contact angles were measured with an OCA-25 optical contact angle measuring instrument (Data Physics Instruments GmbH, Germany). A shale sample was sliced and polished, and the slices were immersed in fresh water, C10-inulin, C12-inulin, or C14-inulin inhibitor solutions for 24 h. The shale slices were then dried at 103 °C for 72 h. Next, 3 μL droplets of deionized water were deposited on the surface of the shale slices; the images of the contact angles of these droplets were saved, and the wettability was analyzed by measuring any changes in these contact angles.

## 4. Conclusions

Three types of acylated inulin, namely C10-inulin, C12-inulin, and C14-inulin, were investigated as potential shale hydration inhibitors (SHIs) in water-based drilling fluids (WBDFs) in this pioneering study. The findings of this research demonstrate that all three acylated inulin variants, particularly C12-inulin and C14-inulin, exhibit outstanding performance in terms of hydration inhibition, surpassing certain conventional SHI options. This enhanced performance can be attributed to two main factors. Firstly, acylated inulin adheres to the shale surface through the formation of hydrogen bonds between the hydrophilic functional groups of inulin and hydroxyl groups. Secondly, the hydrophobic alkyl chain at the other end of acylated inulin forms a compact structure, effectively creating a sealing film that prevents water intrusion. At a concentration of 0.5%, C14-inulin achieves a contact angle of 44.6° on the shale surface. Additionally, acylated inulin reduces the surface tension of water, with 1% C14-inulin exhibiting a surface tension of 20.9 mN/m, thereby impeding water infiltration. Moreover, the acylated inulins are non-toxic and biodegradable, possessing EC_50_ values of ≥30,000 and BOD_5_/COD > 0.1, which meets increasingly stringent environmental regulations. Consequently, the three acylated inulins synthesized in this study exhibit considerable potential as high-performance SHIs in WBDFs, offering novel insights into wellbore stabilization technology for water-based drilling fluids.

## Figures and Tables

**Table 1:** Linear regression equations and EC_50_ values for the ecotoxicological tests.

	Linear Regression Equation	R^2^	EC_50_ (ppm)
C10-inulin	y = 0.0014x + 2.5726	0.9954	33,876.7143
C12-inulin	y = 0.0014x + 4.2571	0.9918	32,673.5
C14-inulin	y = 0.0014x + 7.0988	0.9947	30,643.7143

**Table 2:** COD, BOD_5_, and BOD_5_/COD of 1% C10-inulin, 1% C12-inulin, and 1% C14-inulin.

	Measured Values			Standard Value	Classification
	C10-Inulin	C12-Inulin	C14-Inulin		
BOD_5_ (ppm)	15.4	15.7	15.9	<20	qualified
COD (ppm)	49.6	56.1	58.9	<100	qualified
BOD_5_/COD	0.31	0.28	0.27	0.10	biodegradable

**Table 3:** Mineral composition of the shale samples.

Mineral Composition	Content (%)	Component of Clay Mineral	Relative Content (%)
Quartz	41.3	Kaolinite	1.9
Potassium feldspar	2.1	Illite	29.9
Sodium feldspar	3.8	Chlorite	16.1
Calcite	6.9	Illite/Smectite mixed layer	52.1
Dolomite	4.2		
Clay mineral	41.7		

## Data Availability

Data are contained within the article.

## Nomenclature

OBDFs	oil-based drilling fluids
WBDFs	water-based drilling fluids
SHIs	shale hydration inhibitors
SBSs	sugar-based surfactants
C10	decanoyl chloride
C12	lauroyl chloride
C14	myristoyl chloride
XRD	X-ray diffraction
SEM	scanning electron microscopy
KCl	potassium chloride
PA	poly (ester amine)
Na-bent	sodium-based bentonite
AV	apparent viscosity
PV	plastic viscosity
CST	capillary suction time
CA	contact angle
COD	chemical oxygen demand
BOD	biochemical oxygen demand
EC_50_	concentration for 50% of maximal effect
